# Tilapia Skin Peptides Ameliorate Diabetic Nephropathy in STZ-Induced Diabetic Rats and HG-Induced GMCs by Improving Mitochondrial Dysfunction

**DOI:** 10.3390/md18070363

**Published:** 2020-07-15

**Authors:** Lin Jin, Dongxiao Zheng, Guanyu Yang, Wei Li, Huan Yang, Qian Jiang, Yongjun Chen, Yingxia Zhang, Xi Xie

**Affiliations:** 1 Key Laboratory of Tropical Biological Resources of Ministry of Education, Hainan University, Haikou 570228, China; jinlin12470@hainanu.edu.cn (L.J.); zhengdongxiao@hainanu.edu.cn (D.Z.); yangguanyu@hainanu.edu.cn (G.Y.); zhangyingxia@hainanu.edu.cn (Y.Z.); 2 School of Life and Pharmaceutical Sciences, Hainan University, Haikou 570228, China; jiang123abcc@163.com; 3 School of Materials Science and Engineering, Hainan University, Haikou 570228, China; liv880213@foxmail.com (W.L.); huanhuanyang@hainu.edu.cn (H.Y.); chenyj99@163.com (Y.C.); 4 Key Laboratory of Advanced Materials of Tropical Island Resources, Ministry of Education, Hainan University, Haikou 570228, China

**Keywords:** fish peptides, diabetic nephropathy, lipid metabolism, renal fibrosis, mitochondria, Bnip3/Nix signaling

## Abstract

Diabetic nephropathy (DN) is one of the major microvascular complications of diabetes, and mitochondrial dysfunction has been observed in the kidneys of diabetic patients. Tilapia skin peptides (TSPs) are mixtures of small-molecular-weight peptides derived from tilapia skin. Rising evidence suggests that bioactive peptides from marine sources are beneficial for DN. This study aimed to investigate whether TSPs can alleviate the pathological progress in experimental DN by improving mitochondrial dysfunction through the activation of Bnip3/Nix signaling. In the current study, TSPs treatment alleviated the metabolic parameters and renal morphology in streptozotocin-induced diabetic rats. Additionally, TSPs treatment significantly activated Bnip3/Nix signaling and improved the mitochondrial morphology, reversed the over-production of mitochondrial superoxide and cellular reactive oxygen species and the decreased mitochondrial membrane potential, thereby inhibiting the expressions of fibronectin, collagen IV and intercellular cell adhesion molecule-1 in glomerular mesangial cells induced by high glucose. Collectively, our results suggest that TSPs show the renoprotective effect on DN by improving mitochondrial dysfunction, and they can be a potential therapeutic strategy for DN.

## 1. Introduction

Diabetic nephropathy (DN), one of the major microvascular complications of diabetes, is a globally recognized health disaster [1]. DN is characterized by excessive extracellular matrix (ECM) deposition, glomerular and tubular hypertrophy, glomerular basement membrane thickening, and increase in mesangial matrix, eventually leading to glomerulosclerosis and tubulointerstitial fibrosis [2].

Glomerular mesangial cells (GMCs) are the main functional cells of the kidney. In the case of diabetes mellitus (DM), the ECM components, such as collagen and fibronectin (FN), accumulate in large quantities and the synthesis and expression of profibrotic cytokines increase in GMCs, resulting in the expansion of the mesangial region, mediating the pathological changes in renal fibrosis and eventually leading to end-stage renal disease [3].

Mitochondria are important functional organelles of the body and are the main source of reactive oxygen species (ROS) in hyperglycemic conditions [4]. Excessive ROS in renal tissues will lead to the over-production of FN and intercellular cell adhesion molecule-1 (ICAM-1), which initiates and participates in the pathological process of DN [5]. The stability of mitochondrial internal environment is crucial to the maintenance of renal function, and alleviating mitochondrial dysfunction is an important strategy for DN treatment [6].

Autophagy, a lysosomal degradation pathway, plays a key role in maintaining intracellular homeostasis by removing protein aggregates and damaged or excess organelles [7]. Mitophagy is the selective degradation of mitochondria by autophagy. Defective mitophagy occurred in the glomerulus and tubules of *db/db* mice, while the restoration of mitophagy exerted beneficial effects on DN [8,9]. The perturbation of mitochondrial quality control, including mitochondrial dynamics and autophagy/mitophagy, were observed in renal proximal tubular epithelial cell lines HK-2 (human) and LLC-PK1 (porcine) cells subjected to high-glucose (HG) [10]. Mitochondrial dysfunction was observed in the kidneys of diabetic patients and efficient removal of the accumulated dysfunctional mitochondria through mitophagy could help to ameliorate the pathological process of DN [11,12]. The molecular mechanism of mitophagy involves a variety of interrelated signaling, among which Bnip3/Nix signaling plays a regulatory role [13]. Bnip3 and Nix are known to promote mitophagy as they possess LC3 interacting region (LIR) that interacts with LC3-II [6]. When mitochondria are damaged, mitophagy can remove the damaged mitochondria through Bnip3/Nix signaling. The increased protein levels of Bnip3 were jointly involved in the occurrence of mitophagy [14].

The bioactive peptides derived from marine resources have beneficial effects on several metabolism-related health outcomes [15]. Marine collagen peptides derived from fishes are emerging as a potential therapeutic strategy for type 2 DM due to their antioxidative, angiotensin-I converting enzyme (ACE) inhibitory and dipeptidyl peptidase IV inhibitory activities [16]. In addition, oligopeptides derived from marine fish in the East China Sea benefited glucose and lipid metabolism, insulin sensitivity and renal function in Chinese patients with type 2 DM and hypertension [17]. The tilapia skin peptides (TSPs) used in the present study are mixtures of small-molecular-weight peptides derived from tilapia skin. Furthermore, the by-products of fish processing are an excellent source of high-quality protein, and utilizing marine by-products to extract bioactive substances is environment-friendly and cost-effective, positively affecting the industry and human health [18]. This study aimed to explore whether TSPs can alleviate the pathological injury in experimental DN by protecting mitochondria through activating Bnip3/Nix signaling.

## 2. Results

### 2.1. Effect of TSPs on Metabolic and Renal Parameters in Streptozotocin (STZ)-Induced Diabetic Rats

**Figure 1:**
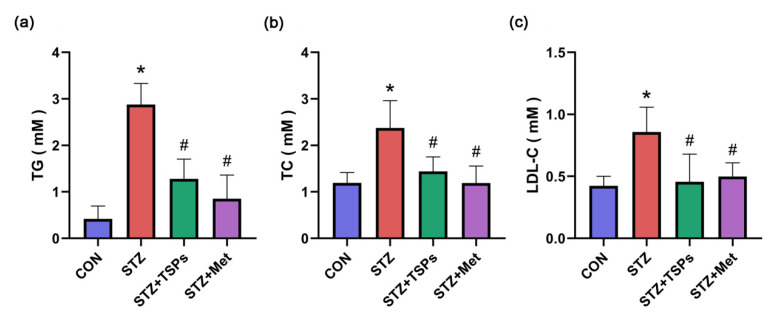
Effects of TSPs on abnormal lipid metabolism in STZ-induced diabetic rats. Serum levels of triacylglycerol (TG) (**a**), total cholesterol (TC) (**b**) and low-density lipoprotein cholesterol (LDL-C) (**c**) in the control, diabetic, TSPs and positive control groups were measured as described in Materials and Methods. * *p* < 0.05 vs. control group, # *p* < 0.05 vs. diabetic group.

The kidney hypertrophy index (KW/BW), fasting blood glucose (FBG), blood urea nitrogen (BUN), serum creatinine (Cr), and urine protein (UP) over 24 h significantly increased in STZ-induced diabetic rats compared with those in the control group (*p* < 0.05, Table 1). After 8 weeks of treatment with TSPs (3 g/kg daily i.g.), the diabetic rats exhibited a significant reduction in these parameters, except for blood glucose (*p* < 0.05; Table 1). After 8 weeks of treatment with metformin (0.23 g/kg daily i.g.), the diabetic rats exhibited a significant reduction in all the parameters (*p* < 0.05; Table 1). Parameters related to lipid metabolism were also detected. Serum triacylglycerol (TG), total cholesterol (TC) and low-density lipoprotein cholesterol (LDL-C) significantly increased in STZ-induced diabetic rats compared with those in the control group (Figure 1). The increased levels of TG, TC and LDL-C in diabetic rats were reduced by TSPs or metformin treatment (Figure 1).

### 2.2. TSPs Improve the Renal Morphology and ECM Accumulation in STZ-Induced Diabetic Rats

**Figure 2:**
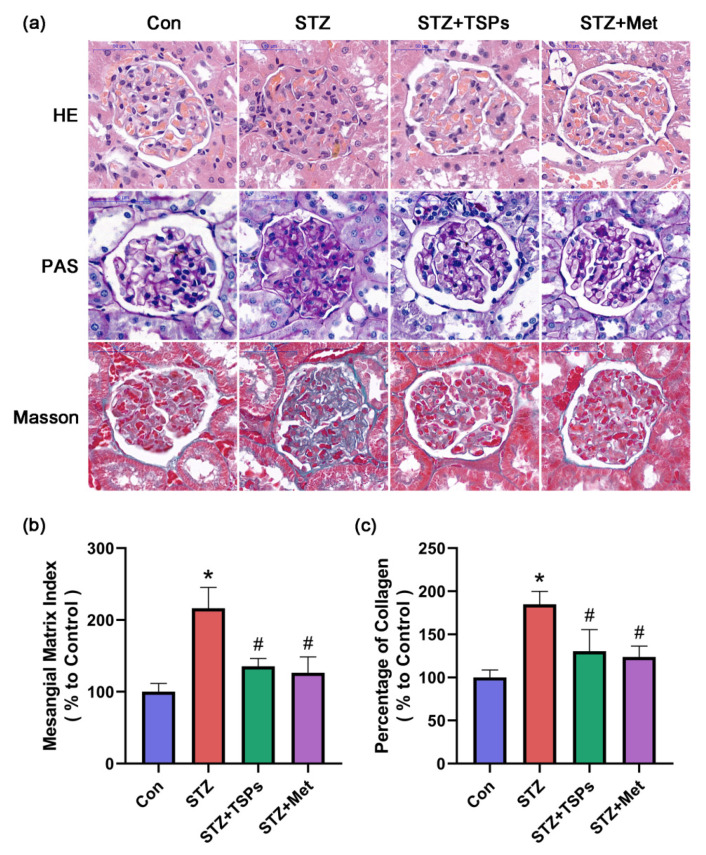
Glomerular injury in the kidneys of STZ-induced diabetic rats. (**a**) Hematoxylin-eosin (HE) and periodic-acid Schiff (PAS) staining were performed to explore glomerular histopathology. Collagen accumulation was assessed by Masson staining (blue indicates collagen). The images present the representative glomeruli of HE-, PAS- and Masson-stained sections in the control, diabetic, TSPs and positive control groups (scale bar = 50 μm). (**b**) Mesangial matrix index (MMI) of glomeruli in the PAS-stained images and (**c**) percentage of collagen in the Masson-stained images were semi-quantified as described in Materials and Methods. * *p* < 0.05 vs. control group, # *p* < 0.05 vs. diabetic group.

After intragastric administration of TSPs (3 g/kg daily i.g.) or metformin (0.23 g/kg daily i.g.) for 8 weeks, renal histological alterations, glycogen deposition, and collagen accumulation in rats were evaluated. Figure 2 shows the representative glomerulus sections with hematoxylin-eosin (HE), periodic-acid Schiff (PAS), and Masson staining. HE staining showed renal damage, including glomerular hypertrophy and matrix expansion, in the diabetic rats. The renal histopathological changes were significantly improved after TSPs or metformin treatment (Figure 2a). In contrast to the control group, a significant increase in PAS-positive mesangial matrix was observed in the glomerulus of STZ-induced diabetic rats. Treatment with TSPs reversed this increase, similarly to the effect on the positive control group (Figure 2a). To evaluate the effect of TSPs on mesangial matrix hyperplasia, we calculated the mesangial matrix index (MMI, in%), which significantly increased in the diabetic group compared with the control group. However, TSPs treatment markedly reduced the MMI of diabetic rats, and the MMI of positive control group was reduced to a level close to that of the control group (Figure 2b). Masson staining revealed an increased collagen accumulation in the glomerulus of STZ-induced diabetic rats. TSPs treatment reduced the excessive deposition of collagen (Figure 2a). The percentage of collagen, which increased in the STZ-induced diabetic group, reduced in TSPs and positive control groups (Figure 2c).

### 2.3. TSPs Prevent the Increased Protein Expressions of FN, Collagen IV and ICAM-1 in GMCs Challenged with HG

**Figure 3:**
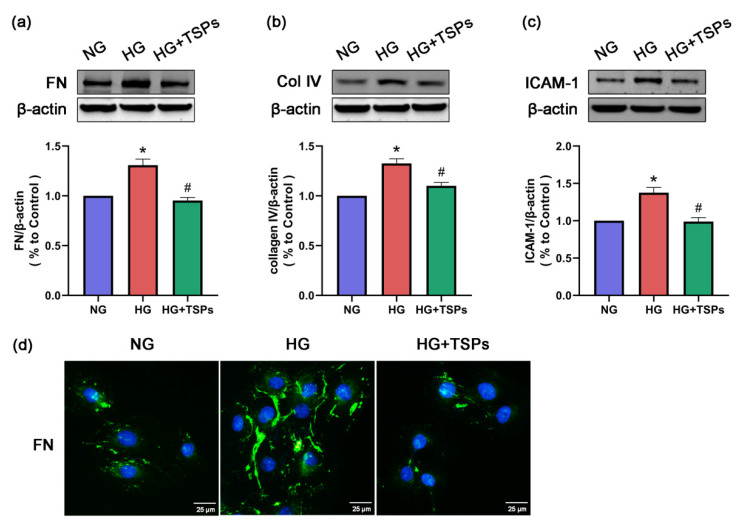
TSPs inhibited high-glucose (HG)-induced protein expressions of fibronectin (FN), collagen IV and intercellular cell adhesion molecule-1 (ICAM-1) in glomerular mesangial cells (GMCs). (**a**–**c**) After 48 h of TSPs (50 μg/mL) treatment of GMCs cultured with HG, we explored the protein expressions of FN, collagen IV and ICAM-1 by Western blot. β-actin was measured as the loading control. All experiments were performed independently at least thrice with similar results. * *p* < 0.05 vs. NG, # *p* < 0.05 vs. HG. (**d**) Immunofluorescence images of FN distribution under the fluorescence microscope. Green indicates localization of FN. Blue represents nuclei. Scale bar = 25 μm.

Given that progressive accumulation of ECM in the glomerular mesangial region is one of the main pathological features of DN, we explored the effect of TSPs on ECM components in vitro. As shown in Figure 3a–c, the expressions of FN, collagen IV and ICAM-1 were upregulated after 48 h of HG treatment. However, TSPs treatment attenuated the increased levels of FN, collagen IV and ICAM-1 induced by HG. In addition, the accumulation of FN was observed by immunofluorescence. Compared with the normal glucose (NG) condition, FN accumulated evidently in GMCs induced by HG. Treatment with TSPs downregulated the HG-induced FN accumulation (Figure 3d). These results indicate that TSPs possess the potential to protect GMCs against HG-induced fibrosis.

### 2.4. TSPs Ameliorate the Excessive Mitochondrial Fragmentation, Decreased Mitochondrial Membrane Potential (MMP), Increased Production of Mitochondrial Superoxide and Cellular ROS in HG-Induced GMCs

**Figure 4:**
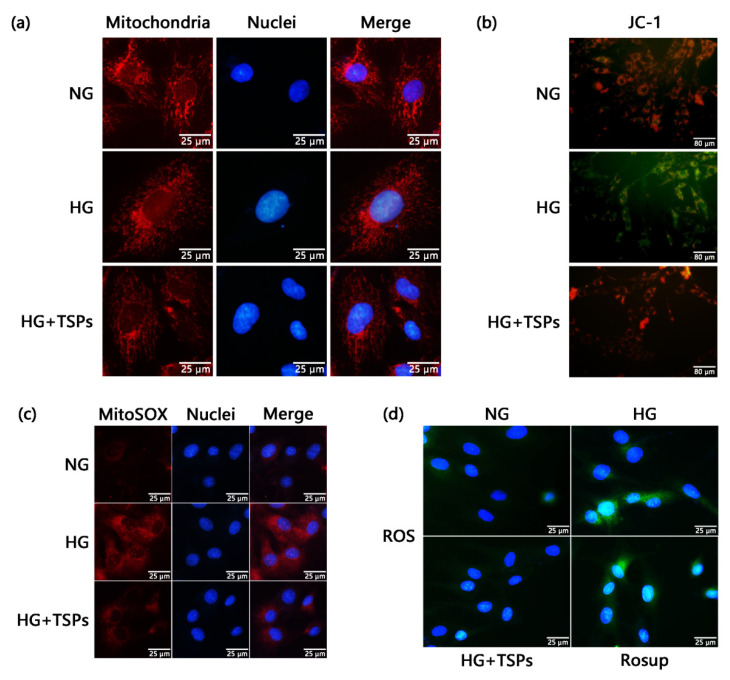
Improvement effects of TSPs on mitochondrial damage and oxidative stress in HG-induced GMCs. (**a**) GMCs were cultured for 48 h of HG treatment with or without TSPs. Fluorescence images stained with Mitotracker Red fluorescent dye (200 μM) were captured using Olympus IX71. Red and blue colors indicate mitochondria and nuclei, respectively. Scale bar = 25 μm. (**b**) GMCs were cultured for 48 h of HG treatment with or without TSPs. Fluorescent probe JC-1 was applied to detect the mitochondrial membrane potential (MMP) levels and the representative fluorescence images were captured using Olympus IX71. Red fluorescence represents JC-1 aggregate and green fluorescence denotes JC-1 monomer. Scale bar = 80 μm. (**c**) GMCs were cultured for 48 h of HG treatment with or without TSPs. Fluorescent probe MitoSOX Red was applied to detect the mitochondrial superoxide levels and the representative fluorescence images were captured using Olympus IX71. Red and blue colors indicate mitochondrial superoxide and nuclei, respectively. Scale bar = 25 μm. (**d**) GMCs were cultured for 48 h of HG treatment with or without TSPs. Rosup (10 μM) was used as a positive reagent to induce reactive oxygen species (ROS) for 30 min. Fluorescent probe DCFH-DA was applied to detect the ROS levels and the fluorescence images stained with DCFH-DA were captured using Olympus IX71. Green indicates ROS and blue represents the nuclei. Scale bar = 25 μm.

Mitochondrial dysfunction and excessive ROS are the main causes of renal fibrosis. We used Mitotracker Red fluorescent dye to observe the mitochondrial morphology of GMCs. As shown in Figure 4a, the mitochondria were elongated and filamentous under the NG condition and were considered to possess normal function. However, mitochondria became short rods of diffused distribution after 48 h of HG treatment. TSPs treatment attenuated the mitochondrial fragmentation induced by HG. We further explored whether mitochondrial functions were impaired by HG treatment. The production levels of mitochondrial superoxide, which was measured by MitoSOX Red (Figure 4c), and ROS, which was measured by 2′,7′-dichlorodihydrofluorescein diacetate (DCFH-DA) (Figure 4d), increased in HG-cultured GMCs, whereas the MMP (Figure 4b) level decreased. However, TSPs treatment significantly reversed the over-production of mitochondrial superoxide and cellular ROS and the decreased MMP in GMCs induced by HG. The fluorescent images suggest that TSPs have improvement effects on mitochondria damage and oxidative stress in HG-induced GMCs.

### 2.5. Bnip3/Nix Signaling is Enhanced by TSPs Treatment in HG-Induced GMCs

**Figure 5:**
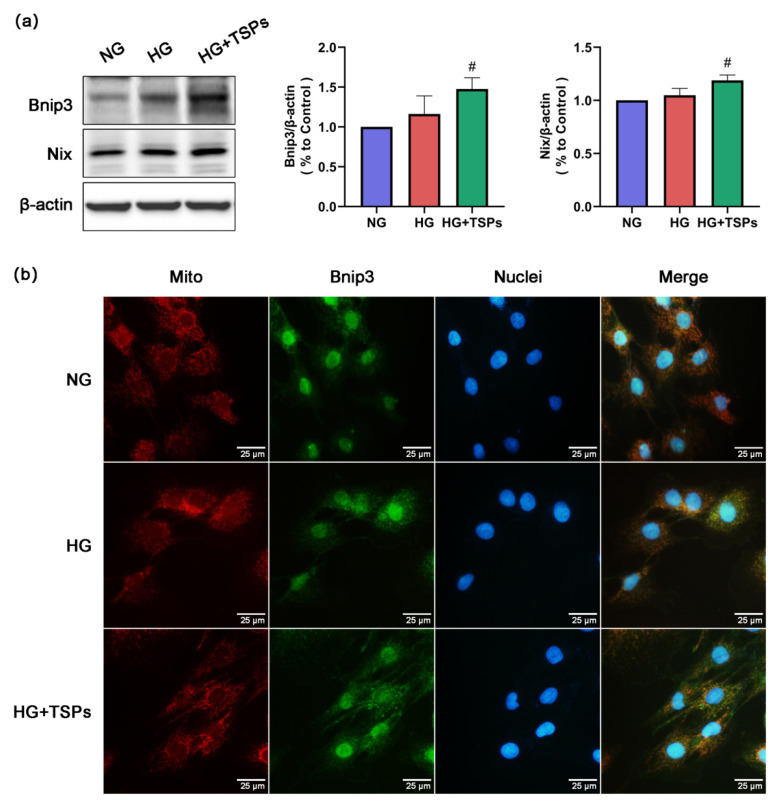
Effects of TSPs on Bnip3/Nix signaling in HG-cultured GMCs. (**a**) After 48 h of TSPs (50 μg/mL) treatment of GMCs cultured in HG, we measured the protein expressions of Bnip3 and Nix by Western blot. β-actin was measured as the loading control. All experiments were performed independently at least thrice with similar results. * *p* < 0.05 vs. NG, # *p* < 0.05 vs. HG. (**b**) Immunofluorescent staining showed the co-localization of mitochondria and Bnip3 (Olympus IX71). The colors red, green, and blue indicate mitochondria, Bnip3 and nuclei, respectively. Scale bar = 25 μm.

To further explore the mechanism of TSPs in protecting mitochondria, we next explored the effect of TSPs on the Bnip3/Nix signaling. The results show that the expressions of Bnip3 and Nix were slightly upregulated in HG-induced GMCs, and TSPs treatment remarkably increased the protein expression levels of Bnip3 and Nix (Figure 5a). Meanwhile, the immunofluorescence results show that the co-localization of Bnip3 and mitochondria increased significantly after TSPs treatment compared with that in HG-induced GMCs (Figure 5b), suggesting that TSPs promote the recruitment of Bnip3 to mitochondria. These results suggest that TSPs can activate the Bnip3/Nix signaling and may have a promoting effect on mitophagy.

## 3. Discussion

In recent decades, DN has become the most common cause of end-stage nephropathy due to the rapid increase in the prevalence of diabetes [19]. With the prevalence of obesity and metabolic syndrome, the incidence of DN will continually increase in the coming decades [20]. No specific drugs are currently available for DN, and the existing treatments are focused on the control of blood glucose and insulin levels [21]. Therefore, studies should explore the strategies for the prevention and treatment of DN. Rising evidence suggests that functional foods containing physiologically active components, either from plants or animal sources, may enhance health [22]. The use of functional foods as complementary therapies for the prevention and management of diseases has steadily increased over the past few decades [23]. Bioactive peptides from natural food sources have an improvement effect on DN. For example, a rapeseed peptide improved the renal function indices and simultaneously attenuated ECM accumulation in DN mice and HG-induced GMCs [24]. Tea polypeptides reduced the levels of FBG, serum insulin level, total urinary protein, Cr, and urine nitrogen in DN mice [25]. Marine peptides possess excellent biological activity to prevent or treat many diseases, including cardiopathy, hypertension, diabetes and obesity [26]. Tilapia skin peptides possessed ACE inhibitory, antioxidant and anti-inflammatory activities [27,28,29]. The tilapia skin collagen polypeptide alleviated liver and kidney injuries in mice induced by D-galactose [30]. The tilapia collagen peptide mixture TY001 improved glucose metabolism and reduced inflammation in STZ-induced diabetic mice [31]. However, a limited number of studies focused on the effect of marine-derived peptides on DN. Here, we speculate that the peptides derived from tilapia skin have the potential to treat DN. Thus, this study aimed to explore whether TSPs have protective effects on DN and further investigate their underlying mechanisms.

In addition to hyperglycemia, hyperlipidemia has been identified as a risk factor for DN; it aggravates glomerular injury by activating multiple signaling pathways [32]. A vicious cycle exists between the development of DN promoted by hyperlipidemia and the dyslipidemia aggravated by diabetes [33]. Therefore, improving abnormal lipid metabolism is a potential strategy to alleviate DN. Peptides from marine fish have been recognized as a dietary supplement that is beneficial to improve lipid metabolism and control blood pressure and blood glucose [34]. Collagen peptide of skate skin reversed the changed plasma levels of TC, LDL-C and high-density lipoprotein to improve lipid metabolism in high-fat fed mice [35]. In the present study, the increased levels of BUN, Cr, UP, TG, TC and LDL-C in STZ-induced diabetic rats were reduced after 8 weeks of TSPs treatment, suggesting that TSPs can improve the renal function and lipid metabolism in diabetic rats. However, the treatment with TSPs for 8 weeks had no effect on the blood glucose levels in STZ-induced diabetic rats, suggesting that the protective effect of TSPs on DN is independent of the regulation of blood glucose. Similarly, a rapeseed peptide improved the renal function indices, including 24-h albuminuria, TC, serum Cr, and BUN levels, but did not lower blood glucose levels in DN mice. Additionally, the rapeseed peptide ameliorated oxidative stress parameters and inhibited the mitogen-activated protein kinase and nuclear factor κB signaling pathways in diabetic mice [24]. We speculated that TSPs might improve the altered metabolic parameters in STZ-induced diabetic rats through alleviating oxidative stress. However, the exact molecular mechanisms need future investigation. Given that TSPs have a good therapeutic effect on renal fibrosis and lipid metabolism, but no hypoglycemic effect on STZ-induced diabetic rats, TSPs will be explored in other fibrosis models and hyperlipidemia models in our subsequent research.

A key pathological characteristic in DN is the accumulation of ECM components, such as FN and collagen IV, in kidney [36]. Hence, alleviating the hyperglycemia-induced excessive accumulation of ECM and fibrosis can prevent and treat DN. ICAM-1 is a major downstream inflammatory factor and ICAM-1 gene deficiency had an improvement effect on DN in mice models, indicating that ICAM-1 contributes to the pathogenesis of DN [37]. Peptides isolated from *Eucheuma*, an edible seaweed, had a inhibition effect on bleomycin-induced idiopathic pulmonary fibrosis in mice [38]. In this study, the protein levels of FN, collagen IV and ICAM-1 increased in HG-treated GMCs, whereas TSPs treatment significantly reversed these changes. Histopathological analysis revealed the excessive accumulation of ECM and structural pathological damage in kidneys of STZ-induced diabetic rats. However, treatment with TSPs alleviated these histopathological changes, indicating that TSPs can ameliorate the fibrosis and maintain renal morphology by alleviating the ECM accumulation in DN.

Mitochondria are key organelles in controlling cell metabolism and maintaining the body’s physiological functions [39]. Disorders in mitochondrial homeostasis are central to the pathogenesis of DN [40]. Abnormalities of mitochondrial function in the kidney have long been observed in the context of experimental diabetes [41]. One study showed significant mitochondrial fission in HG-induced GMCs under electron microscopy [42]. The MMP level decreased and ROS production increased when mitochondrial dynamic balance was disordered, especially in a divided state [43]. The increased renal mitochondrial ROS production was observed in DN animal models [40]. The study also exhibited that the phenomena of excessive mitochondrial division, increased ROS production, and upregulated expression of collagen IV in HG-induced GMCs were inhibited by Mdivi-1, which is an inhibitor of mitochondrial division [42]. Interestingly, peptides from marine sources exhibit mitochondrial protective effects. The *Meretrix meretrix* oligopeptides ameliorated the abnormal mitochondrial morphology and enhanced the antioxidant capacity of liver tissue in high-fat diet-fed mice [44]. Low-molecular-weight oligopeptides isolated from sea cucumber had a decreasing effect on the elevated levels of serum ROS in *db/db* mice [45]. In this study, the increase in mitochondrial superoxide and ROS, reduction in MMP and excessive mitochondrial fragmentation in HG-induced GMCs were reversed after TSPs treatment, indicating that TSPs possess a protective effect on mitochondrial damage, and thereby reduce ROS production and alleviate fibrosis in GMCs cultured with HG. Goby fish protein hydrolysates showed amelioration effects on the high blood glucose and oxidative status, structure and function of kidney in high-fat-high-fructose diet-induced rats [46]. Interestingly, given the specificity of the enzyme preparations, the two hydrolysates differed in terms of amino acid combinations and showed varied effects on relieving oxidative stress in hyperglycemia. We speculate that the protective effect of TSPs on DN is partially dependent on antioxidant action of marine-derived specific amino acid sequence. Thus, further investigations are required to determine the amino acid sequences of TSPs that confer protective effect on DN.

Improving damaged mitophagy may be a strategy for alleviating DN. Coenzyme Q10, as an effective antioxidant in mitochondria, exerts beneficial effects on DN through restoring mitophagy [8]. Promoting mitophagy relieved cellular senescence in HG-induced renal tubular epithelial cells. Bnip3, a member of the Bcl-2 family, mainly distributes in the mitochondrial membrane. Bnip3 is a mitophagy receptor and plays a role in promoting survival in certain pathological conditions [47]. Nix is also a typical autophagy receptor with an n-terminal LIR motif exposed to the cytoplasm, which can introduce mitochondria into autophagosomes through direct interaction with Atg8 family proteins [13]. Bnip3 and Nix are homologous, and both are important molecular mediators of mitophagy. Trehalose administration restored disrupted autophagic flux and mitophagy by activating Bnip3 and increasing the co-localization of Bnip3 with mitochondria in a mouse osteoarthritis model [48]. In the nonalcoholic fatty liver model, the Bnip3 signaling played a key role in alleviating mitochondrial injury and promoting mitophagy [49]. *Panax notoginseng* saponins mitigated cisplatin-induced nephrotoxicity by enhancing mitophagy via a hypoxia-inducible factor-1α/Bnip3/Beclin-1 signaling [50]. The restoration of Nix-mediated mitophagy might be a novel therapeutic target for alleviating proteinuria-induced kidney injury [51]. In our study, the expressions of Bnip3 and Nix were slightly elevated in GMCs cultured with HG, suggesting that GMCs may sense the HG-induced injury and attempt to activate mitophagy. However, the effort was insufficient to relieve mitochondrial damage. Therefore, we hypothesized that TSPs may enhance mitophagy activity by activating the Bnip3/Nix signaling to protect mitochondria through relieving the accumulation of damaged mitochondria, eventually alleviating fibrosis in HG-induced GMC. Consistent with our speculation, after TSPs treatment, the protein expressions of Bnip3 and Nix were significantly upregulated, and the recruitment of Bnip3 to mitochondria was significantly increased.

## 4. Materials and Methods

### 4.1. Reagents and Materials

NG Dulbecco’s modified Eagle medium (DMEM) and HG DMEM were obtained from Gibco (New York, NY, USA). STZ was purchased from Sigma-Aldrich (St. Louis, MO, USA). TSPs were obtained from Hainan Hai Xian Bao Biological Technology Co., Ltd. (Haikou, China). The bicinchoninic acid protein assay kit, BeyoECL Plus, ROS assay kit and mitochondrial membrane potential assay kit with JC-1 were obtained from Beyotime (Haimen, China). Hoechst 33342 stain solution, MitoTracker Red CMXRos and MitoSOX Red mitochondrial superoxide indicator were obtained from Yeasen (Shanghai, China). The antibodies used in our study included antibodies against FN (Abcam, Cambridge, UK); collagen IV, ICAM-1, and β-actin (Boster, Wuhan, China); Bnip3 and Nix (Solarbio, Beijing, China); and Alexa Fluor 488 (Invitrogen, Carlsbad, CA, USA).

### 4.2. Animal Experiment

Male Sprague–Dawley rats (*n* = 32, 200 ± 10 g) were supplied by the Medical Experimental Animal Center of Guangdong Province (Guangzhou, China) with the permit 44007200065142. All animal procedures conformed to China Animal Welfare Legislation with efforts made to minimize the number of animals and their discomfort. All animals were housed under standard conditions with free access to food and water. After feeding with a regular diet for 1 week, the rats were assigned to a diabetic model group (*n* = 24), which was fed with a high-fat, HG diet for the following 4 weeks, and a normal control group (*n* = 8), which were fed with a normal diet. After 4 weeks of dietary manipulation, the diabetic model group rats were given a single intraperitoneal injection of freshly prepared STZ (30 mg/kg). The normal control group rats were injected with an equal volume of citrate buffer. At 72 h after injection, rats with an FBG of 11.1 mmol/L and above were considered as diabetic rats. The diabetic rats were randomized into the administration group (*n* = 8) to receive TSPs (3 g/kg daily i.g.) and positive control group (*n* = 8) to receive metformin (0.23 g/kg daily i.g.); the other diabetic rats received an equal volume of distilled water. Rats were sacrificed after 8 weeks of treatment. The dose of TSPs treatment was in accordance with a previous animal study [52] and metformin was calculated based on body surface area and clinical dose of the drug. Blood sample was collected from the abdominal vein and serum was obtained by centrifugation at 3000× *g* for 15 min and stored at −80 °C or fixed in 10% neutral buffered formalin.

### 4.3. Biochemical and Morphological Studies

Blood glucose, BUN, serum Cr, UP, TG, TC, and LDL-C were analyzed in Servicebio Technology (Wuhan, China). BUN was tested with UREA/BUN assay kit (Changchun Huili Biotech, Changchun, China), serum Cr was measured with Cr assay kit (Changchun Huili Biotech, Changchun, China), UP was determined with UP assay kit (Nanjing Jiancheng Bioengineering Institute, Nanjing, China), TG was tested with the TG assay kit (Rayto, Shenzhen, China), TC was measured with cholesterol assay kit (Rayto, Shenzhen, China), and LDL-C was determined with LDL assay kit (Rayto, Shenzhen, China) following the manufacturer’s instructions. These parameters were analyzed with Rayto Chemray 240 Automated Chemistry Analyzer and Rayto Chemray 800 Automated Chemistry Analyzer (Rayto, Shenzhen, China). For histological examination, the cortexes of kidneys were separated, fixed in 10% formaldehyde, and embedded in paraffin. Standard sections of 4 μm thickness were cut. HE, PAS, and Masson staining were used for standard sections. The cross section of the glomerulus with its maximum diameter was photographed. Forty glomeruli were randomly selected from the three slides of each animal before the analysis by Image-Pro Plus. The MMI was calculated as the ratio of the mesangial area to the glomerular area ×100. Masson staining was performed to evaluate collagen expression.

### 4.4. Cell Culture and Treatment

The rat GMCs were obtained from Sun Yat-sen University (Guangzhou, China). The GMCs used in our study were within 10th passages. The confluent GMCs were incubated in serum-free DMEM for 24 h before the treatment with NG (5.6 mM) or HG (30 mM) mixed with TSPs at various times.

### 4.5. Western Blot Assay

The proteins were extracted using radioimmunoprecipitation assay with protease inhibitor phenylmethylsulfonyl fluoride. After incubation for 15 min on ice, 30 min of 12,000× *g* centrifugation at 4 °C was performed to obtain the supernatant, and protein concentration was quantified by BCA protein assay kit. The proteins were separated by 8–15% sodium dodecyl sulfate-polyacrylamide gel electrophoresis, and transferred to nitrocellulose membrane (Bio-Rad Laboratories, Hercules, CA, USA). After blocking with 5% skim milk in Tris-buffered saline, 0.1% Tween 20 for 1 h at room temperature, the membrane was incubated with corresponding antibodies for 12 h at 4 °C and subsequently incubated with horseradish peroxidase-conjugated secondary antibodies for 1 h at room temperature. Through BeyoECL Plus, immunoreactive bands were detected by the Gel Image System (JunYi, Beijing, China), and the gray value of bands were analyzed by ImageJ. β-actin was set as the loading control.

### 4.6. Immunofluorescence

After treatment with the corresponding stimuli, the cells were washed with phosphate-buffered saline (PBS), fixed with 4% paraformaldehyde in PBS for 20 min and permeabilized using 0.1% Triton X-100 in PBS for another 10 min at room temperature. After blocking with goat serum for 30 min at room temperature, the cells were incubated with primary antibodies, including rabbit antibody against FN and Bnip3, in 10% goat serum overnight at 4 °C. Coverslips were washed with PBS thrice and then incubated with Alexa Fluor 488-conjugated secondary antibody in the dark for 1 h at room temperature. The nuclei were co-labeled with Hoechst 33342 stain solution for 15 min at room temperature. Finally, the coverslips were mounted on slides with antifade mounting medium. The images were collected using a fluorescence microscope (Olympus, Tokyo, Japan).

### 4.7. Assessment of Mitochondrial Morphology

The mitochondrial morphology was assessed by MitoTracker Red CMXRos. The Mitotracker probe was diluted to 250 μM with serum-free DMEM. After incubation with the Mitotracker staining working solution at 37 °C for 30 min and washing by PBS thrice, the cells were observed with a fluorescence microscope (Olympus, Tokyo, Japan).

### 4.8. Measurement of Mitochondrial Superoxide

The mitochondrial superoxide levels were measured by MitoSOX Red mitochondrial superoxide indicator. MitoSOX probe was diluted to 5 μM with Hank’s balanced salt solution (HBSS). After incubation with MitoSOX staining working solution at 37 °C for 10 min and washing by HBSS thrice, the cells were observed with fluorescence microscope (Olympus, Tokyo, Japan).

### 4.9. Measurement of ROS

The ROS production was measured by ROS assay kit. Rosup (10 μM) was used as a positive reagent to induce ROS for 30 min. After incubation with fluorescent probe DCFH-DA (10 μM) at 37 °C for 20 min and washing by PBS thrice, the cells were incubated with Hoechst 33342 (20 μM) at 37 °C for 10 min and washed again by PBS thrice. Then, the cells were observed with fluorescence microscope (Olympus, Tokyo, Japan).

### 4.10. Measurement of MMP

The MMP levels were measured by mitochondrial membrane potential assay kit with JC-1. After incubation with 1 × JC-1 staining working solution at 37 °C for 20 min and washing by 1 × staining buffer twice, the cells were observed with fluorescence microscope (Olympus, Tokyo, Japan).

### 4.11. Statistical Analysis

All experiments were performed independently at least three times with similar results. The data were analyzed using SPSS 26.0 software and the values were expressed as the means ± SD. Statistical analyses of data were performed by one-way analysis of variance using post hoc multiple comparisons. *p* < 0.05 was considered statistically significant.

## 5. Conclusions

In conclusion, our study demonstrated that TSPs treatment significantly improved the pathological damages of experimental DN, including renal function, histopathological changes and lipid metabolism. The protective effect of TSPs on renal fibrosis in DN partly depends on the regulation of mitophagy via Bnip3/Nix signaling to improve mitochondrial dysfunction, including mitochondrial fragmentation, the decreased MMP and over-production of mitochondrial superoxide and cellular ROS. Based on the limitation of the present study, our future work will explore the metabolism of TSPs in vivo.

## Figures and Tables

**Table 1:** Effects of tilapia skin peptides (TSPs) on renal metabolic and biochemical parameters in streptozotocin (STZ)-induced diabetic rats.

Parameters	Control (*n* = 8)	STZ (*n* = 8)	STZ + TSPs (*n* = 8)	STZ + Met (*n* = 8)
KW/BW (%)	0.40 ± 0.02 #	0.80 ± 0.67 *	0.69 ± 0.09 *,#	0.69 ± 0.10 *,#
FBG (mM)	4.27 ± 0.15 #	22.18 ± 2.48 *	19.63 ± 1.43 *	8.83 ± 2.08 *,#
BUN (mM)	8.39 ± 0.33 #	22.06 ± 1.22 *	18.34 ± 1.05 *,#	16.77 ± 4.33 *,#
Cr (μM)	21.84 ± 8.70 #	89.66 ± 12.45 *	65.64 ± 10.77 *,#	50.98 ± 20.23 *,#
UP 24 h (mg)	9.65 ± 3.98 #	29.62 ± 8.53 *	14.04 ± 4.94 #	12.77 ± 6.46 #

KW/BW: kidney hypertrophy index, FBG: fasting blood glucose, BUN: blood urea nitrogen, Cr: serum creatinine, UP 24 h: urine protein for 24 h, Data are means ± standard deviation (SD), *n* = 8. * *p* < 0.05 vs. control group, # *p* < 0.05 vs. STZ-induced diabetic group.

## References

[1] Huang K., Chen C., Hao J., Huang J., Wang S., Liu P., Huang H. (2015). Polydatin promotes Nrf2-ARE anti-oxidative pathway through activating Sirt1 to resist AGEs-induced upregulation of fibronetin and transforming growth factor-beta1 in rat glomerular messangial cells. Mol. Cell. Endocrinol..

[2] Shah I.M., Mackay S.P., McKay G.A. (2009). Therapeutic strategies in the treatment of diabetic nephropathy—A translational medicine approach. Curr. Med. Chem..

[3] Chen Z., Xie X., Huang J., Gong W., Zhu X., Chen Q., Huang J., Huang H. (2017). Connexin43 regulates high glucose-induced expression of fibronectin, ICAM-1 and TGF-β1 via Nrf2/ARE pathway in glomerular mesangial cells. Free Radic. Biol. Med..

[4] Yu T., Robotham J.L., Yoon Y. (2006). Increased production of reactive oxygen species in hyperglycemic conditions requires dynamic change of mitochondrial morphology. Proc. Natl. Acad. Sci. USA.

[5] Xie X., Chen Q., Tao J. (2018). Astaxanthin Promotes Nrf2/ARE Signaling to Inhibit HG-Induced Renal Fibrosis in GMCs. Mar. Drugs.

[6] Higgins G.C., Coughlan M.T. (2014). Mitochondrial dysfunction and mitophagy: The beginning and end to diabetic nephropathy?. Br. J. Pharmacol..

[7] Kroemer G., Marino G., Levine B. (2010). Autophagy and the integrated stress response. Mol. Cell.

[8] Xiao L., Xu X., Zhang F., Wang M., Xu Y., Tang D., Wang J., Qin Y., Liu Y., Tang C. (2017). The mitochondria-targeted antioxidant MitoQ ameliorated tubular injury mediated by mitophagy in diabetic kidney disease via Nrf2/PINK1. Redox Biol..

[9] Sun J., Zhu H., Wang X., Gao Q., Li Z., Huang H. (2019). CoQ10 ameliorates mitochondrial dysfunction in diabetic nephropathy through mitophagy. J. Endocrinol..

[10] Zhan M., Usman I.M., Sun L., Kanwar Y.S. (2015). Disruption of renal tubular mitochondrial quality control by Myo-inositol oxygenase in diabetic kidney disease. J. Am. Soc. Nephrol..

[11] Ma Y., Chen Z., Tao Y., Zhu J., Yang H., Liang W., Ding G. (2019). Increased mitochondrial fission of glomerular podocytes in diabetic nephropathy. Endocr. Connect..

[12] Czajka A., Ajaz S., Gnudi L., Parsade C.K., Jones P., Reid F., Malik A.N. (2015). Altered Mitochondrial Function, Mitochondrial DNA and Reduced Metabolic Flexibility in Patients with Diabetic Nephropathy. EBioMedicine.

[13] Novak I., Kirkin V., McEwan D.G., Zhang J., Wild P., Rozenknop A., Rogov V., Löhr F., Popovic D., Occhipinti A. (2010). Nix is a selective autophagy receptor for mitochondrial clearance. EMBO Rep..

[14] Matsushima M., Fujiwara T., Takahashi E., Minaguchi T., Eguchi Y., Tsujimoto Y., Suzumori K., Nakamura Y. (1998). Isolation, mapping, and functional analysis of a novel human cDNA (BNIP3L) encoding a protein homologous to human NIP3. Gene Chromosome Cancer.

[15] Dale H.F., Madsen L., Lied G.A. (2019). Fish–derived proteins and their potential to improve human health. Nutr. Rev..

[16] Xia E.Q., Zhu S.S., He M.J., Luo F., Fu C.Z., Zou T.B. (2017). Marine Peptides as Potential Agents for the Management of Type 2 Diabetes Mellitus—A Prospect. Mar. Drugs.

[17] Zhu C.F., Li G.Z., Peng H.B., Zhang F., Chen Y., Li Y. (2010). Therapeutic effects of marine collagen peptides on Chinese patients with type 2 diabetes mellitus and primary hypertension. Am. J. Med. Sci..

[18] Le Gouic A.V., Harnedy P.A., FitzGerald R.J., Mérillon J.M., Ramawat K.G. (2019). Bioactive peptides from fish protein by-products. Bioactive Molecules in Food.

[19] Jha V., Garcia-Garcia G., Iseki K., Li Z., Naicker S., Plattner B., Saran R., Wang A.Y., Yang C.W. (2013). Chronic kidney disease: Global dimension and perspectives. Lancet.

[20] Alberti K.G., Zimmet P. (2013). Global burden of disease—Where does diabetes mellitus fit in?. Nat. Rev. Endocrinol..

[21] Dong L., Li Y., Zhang D., Zhang H., Han J., Wang Z., Zhou J., Lu C., Su X. (2018). Dietary *Apostichopus japonicus* alleviates diabetes symptoms and modulates genes expression in Kidney Tissues of db/db Mice. J. Agric. Food Chem..

[22] Gul K., Singh A.K., Jabeen R. (2016). Nutraceuticals and functional foods: The foods for the future world. Crit. Rev. Food Sci. Nutr..

[23] Alkhatib A., Tsang C., Tiss A., Bahorun T., Arefanian H., Barake R., Khadir A., Tuomilehto J. (2017). Functional foods and lifestyle approaches for diabetes prevention and management. Nutrients.

[24] Zhang M., Yan Z., Bu L., An C., Wang D., Liu X., Zhang J., Yang W., Deng B., Xie J. (2018). Rapeseed protein-derived antioxidant peptide RAP alleviates renal fibrosis through MAPK/NF-κB signaling pathways in diabetic nephropathy. Drug Des. Dev. Ther..

[25] Deng X., Sun L., Lai X., Xiang L., Li Q., Zhang W., Zhang L., Sun S. (2018). Tea polypeptide ameliorates diabetic nephropathy through RAGE and NF-κB signaling pathway in type 2 diabetes mice. J. Agric. Food Chem..

[26] Pangestuti R., Kim S.K. (2017). Bioactive peptide of marine origin for the prevention and treatment of non-communicable diseases. Mar. Drugs.

[27] Lin H.C., Alashi A.M., Aluko R.E., Sun Pan B., Chang Y.W. (2017). Antihypertensive properties of tilapia (*Oreochromis* spp.) frame and skin enzymatic protein hydrolysates. Food Nutr. Res..

[28] Xiao Z., Liang P., Chen J., Chen M.F., Gong F., Li C., Zhou C., Hong P., Yang P., Qian Z.J. (2019). A peptide YGDEY from *Tilapia* gelatin Hydrolysates inhibits UVB-mediated skin Photoaging by regulating MMP-1 and MMP-9 expression in HaCaT cells. Photochem. Photobiol..

[29] Mei F., Liu J., Wu J., Duan Z., Chen M., Meng K., Chen S., Shen X., Xia G., Zhao M. (2020). Collagen peptides isolated from *Salmo salar* and *Tilapia nilotica* skin accelerate wound healing by altering cutaneous Microbiome colonization via upregulated NOD2 and BD14. J. Agric. Food Chem..

[30] Li D.D., Li W.J., Kong S.Z., Li S.D., Guo J.Q., Guo M.H., Cai T.T., Li N., Chen R.Z., Luo R.Q. (2019). Protective effects of collagen polypeptide from tilapia skin against injuries to the liver and kidneys of mice induced by d-galactose. Biomed. Pharmacother..

[31] Xiong X., Liang J., Xu Y., Liu J., Liu Y. (2020). The wound healing effects of the *Tilapia* collagen peptide mixture TY001 in streptozotocin diabetic mice. J. Sci. Food Agric..

[32] Sacks F.M., Hermans M.P., Fioretto P., Valensi P., Davis T., Horton E., Wanner C., Al-Rubeaan K., Aronson R., Barzon I. (2014). Association between plasma triglycerides and high-density lipoprotein cholesterol and microvascular kidney disease and retinopathy in type 2 diabetes mellitus: A global case-control study in 13 countries. Circulation.

[33] Kreisberg R.A. (1998). Diabetic dyslipidemia. Am. J. Cardiol..

[34] Tak Y.J., Kim Y.J., Lee J.G., Yi Y.-H., Cho Y.H., Kang G.H., Lee S.Y. (2019). Effect of oral ingestion of low-molecular collagen peptides derived from skate (*Raja Kenojei*) skin on body fat in overweight adults: A randomized, double-blind, placebo-controlled trial. Mar. Drugs.

[35] Woo M., Song Y.O., Kang K.H., Noh J.S. (2018). Anti-obesity effects of collagen peptide derived from skate (*Raja kenojei*) skin through regulation of lipid metabolism. Mar. Drugs.

[36] Kolset S.O., Reinholt F.P., Jenssen T. (2012). Diabetic nephropathy and extracellular matrix. J. Histochem. Cytochem..

[37] Donate-Correa J., Luis-Rodríguez D., Martín-Núñez E., Tagua V.G., Hernández-Carballo C., Ferri C., Rodríguez-Rodríguez A.E., Mora-Fernández C., Navarro-González J.F. (2020). Inflammatory targets in diabetic nephropathy. J. Clin. Med..

[38] Yu H., Zhang Z., Huang H., Wang Y., Lin B., Wu S., Ma J., Chen B., He Z., Wu J. (2019). Inhibition of bleomycin-induced pulmonary fibrosis in mice by the novel peptide EZY-1 purified from *Eucheuma*. Food Funct..

[39] Porporato P.E., Payen V.L., Baselet B., Sonveaux P. (2016). Metabolic changes associated with tumor metastasis, part 2: Mitochondria, lipid and amino acid metabolism. Cell. Mol. Life Sci..

[40] Coughlan M.T., Thorburn D.R., Penfold S.A., Laskowski A., Harcourt B.E., Sourris K.C., Tan A.L., Fukami K., Thallas-Bonke V., Nawroth P.P. (2009). RAGE-induced cytosolic ROS promote mitochondrial superoxide generation in diabetes. J. Am. Soc. Nephrol..

[41] Rosca M.G., Mustata T.G., Kinter M.T., Ozdemir A.M., Kern T.S., Szweda L.I., Brownlee M., Monnier V.M., Weiss M.F. (2005). Glycation of mitochondrial proteins from diabetic rat kidney is associated with excess superoxide formation. Am. J. Physiol.-Ren. Physiol..

[42] Zhang L., Ji L., Tang X., Chen X., Li Z., Mi X., Yang L. (2015). Inhibition to DRP1 translocation can mitigate p38 MAPK-signaling pathway activation in GMC induced by hyperglycemia. Ren. Fail..

[43] Ayanga B.A., Badal S.S., Wang Y., Galvan D.L., Chang B.H., Schumacker P.T., Danesh F.R. (2016). Dynamin-related protein 1 deficiency improves mitochondrial fitness and protects against progression of diabetic nephropathy. J. Am. Soc. Nephrol..

[44] Huang F., Wang J., Yu F., Tang Y., Ding G., Yang Z., Sun Y. (2018). Protective effect of *Meretrix meretrix* Oligopeptides on high-fat-diet-induced non-alcoholic fatty liver disease in mice. Mar. Drugs.

[45] Li D., Li L., Xu T., Wang T., Ren J., Liu X., Li Y. (2018). Effect of low molecular weight Oligopeptides isolated from sea cucumber on diabetic wound healing in db/db mice. Mar. Drugs.

[46] Nasri R., Abdelhedi O., Jemil I., Daoued I., Hamden K., Kallel C., Elfeki A., Lamri-Senhadji M., Boualga A., Nasri M. (2015). Ameliorating effects of goby fish protein hydrolysates on high-fat-high-fructose diet-induced hyperglycemia, oxidative stress and deterioration of kidney function in rats. Chem. Biol. Interact..

[47] Tang C., Han H., Liu Z., Liu Y., Yin L., Cai J., He L., Liu Y., Chen G., Zhang Z. (2019). Activation of BNIP3-mediated mitophagy protects against renal ischemia-reperfusion injury. Cell Death Dis..

[48] Tang Q., Zheng G., Feng Z., Chen Y., Lou Y., Wang C., Zhang X., Zhang Y., Xu H., Shang P. (2017). Trehalose ameliorates oxidative stress-mediated mitochondrial dysfunction and ER stress via selective autophagy stimulation and autophagic flux restoration in osteoarthritis development. Cell Death Dis..

[49] Li R., Xin T., Li D., Wang C., Zhu H., Zhou H. (2018). Therapeutic effect of Sirtuin 3 on ameliorating nonalcoholic fatty liver disease: The role of the ERK-CREB pathway and Bnip3-mediated mitophagy. Redox Biol..

[50] Liang X., Yang Y., Huang Z., Zhou J., Li Y., Zhong X. (2017). Panax notoginseng saponins mitigate cisplatin induced nephrotoxicity by inducing mitophagy via HIF-1α. Oncotarget.

[51] Xu D., Chen P., Wang B., Wang Y., Miao N., Yin F., Cheng Q., Zhou Z., Xie H., Zhou L. (2019). NIX-mediated mitophagy protects against proteinuria-induced tubular cell apoptosis and renal injury. Am. J. Physiol.-Ren. Physiol..

[52] Zhu C.F., Peng H.B., Liu G.Q., Zhang F., Li Y. (2010). Beneficial effects of oligopeptides from marine salmon skin in a rat model of type 2 diabetes. Nutrition.

